# Novel deep learning-based transcriptome data analysis for drug-drug interaction prediction with an application in diabetes

**DOI:** 10.1186/s12859-021-04241-1

**Published:** 2021-06-11

**Authors:** Qichao Luo, Shenglong Mo, Yunfei Xue, Xiangzhou Zhang, Yuliang Gu, Lijuan Wu, Jia Zhang, Linyan Sun, Mei Liu, Yong Hu

**Affiliations:** 1 Big Data Decision Institute, Jinan University, Guangzhou, 510632 China; 2 School of Management, Jinan University, Guangzhou, 510632 China; 3 Department of Geriatrics, The First Affiliated Hospital of Chongqing Medical University, Chongqing, 400016 China; 4 Xi’an Hospital of Traditional Chinese Medicine, Xi’an, 710021 China; 5 Division of Medical Informatics, Department of Internal Medicine, Medical Center, University of Kansas, Kansas City, KS 66160 USA

**Keywords:** Drug, Drug interaction, Drug safety, Adverse drug event, Deep learning, L1000 database, Transcriptome data analysis

## Abstract

**Background:**

Drug-drug interaction (DDI) is a serious public health issue. The L1000 database of the LINCS project has collected millions of genome-wide expressions induced by 20,000 small molecular compounds on 72 cell lines. Whether this unified and comprehensive transcriptome data resource can be used to build a better DDI prediction model is still unclear. Therefore, we developed and validated a novel deep learning model for predicting DDI using 89,970 known DDIs extracted from the DrugBank database (version 5.1.4).

**Results:**

The proposed model consists of a graph convolutional autoencoder network (GCAN) for embedding drug-induced transcriptome data from the L1000 database of the LINCS project; and a long short-term memory (LSTM) for DDI prediction. Comparative evaluation of various machine learning methods demonstrated the superior performance of our proposed model for DDI prediction. Many of our predicted DDIs were revealed in the latest DrugBank database (version 5.1.7). In the case study, we predicted drugs interacting with *sulfonylureas* to cause hypoglycemia and drugs interacting with *metformin* to cause lactic acidosis, and showed both to induce effects on the proteins involved in the metabolic mechanism in vivo.

**Conclusions:**

The proposed deep learning model can accelerate the discovery of new DDIs. It can support future clinical research for safer and more effective drug co-prescription.

**Supplementary Information:**

The online version contains supplementary material available at 10.1186/s12859-021-04241-1.

## Background

Combination drug therapy is increasingly used to manage complex diseases such as diabetes, cancer, and cardiovascular diseases. In particular, patients with type 2 diabetes often do not only suffer from symptoms of elevated blood glucose levels but also have several comorbidities that require multifactorial pharmacotherapy. Older patients may receive 10 or more concomitant drugs to manage multiple disorders [1, 2]. However, the usage of concomitant drug significantly increases the risk of harm associated with drug-drug interaction (DDI), doubling for each additional drug prescribed [3–7]. DDIs are the major cause of adverse drug events (ADEs) [8, 9], accounting for 20–30% of ADEs [10], and one of the leading reasons for drug withdrawal from the market [11]. DDIs can induce clinical consequences ranging from diminished therapeutic effect to excessive response or toxicity as a result of pharmacokinetics, pharmacodynamics, or a combination of the mechanism [12]. Adverse effects from DDIs may not be recognized until a large cohort of patients has been exposed to clinical practices due to limitations of the in vivo and in vitro models used during the pre-marketing safety screen. As a result, advanced computational methods to predict future DDIs are crucial to reducing unnecessary ADEs.

Over the past decade, deep learning has achieved remarkable success in a number of research areas [13]. Because of its ability to learn at higher levels of abstraction, deep learning has become a promising and effective tool for working with biological and chemical data [14]. Some deep learning techniques have been applied to predict DDI, and significantly improved the prediction accuracy. For example, Ryu et al. proposed DeepDDI, a computation model that predicts DDI with a combination of the structural similarity profile generation pipeline and deep neural network (DNN) [15]. Lee et al*.* built the same DNN model but included three types of features as input: structural similarity profiles, Gene Ontology term similarity profiles, and target gene similarity profiles of known drug pairs; and used autoencoder to reduce the dimensions of each profile [16]. Rohani and Eslahchi developed a neural network-based method with the input of the model being an integrated similarity profile of various information about drug pairs by a non-linear similarity fusion method called SNF [17]. Compared with Random Forest, K-nearest neighbor, and support vector machine, the DNN used in those models shows better performance in DDI prediction [15–17]. Karim et al. used LSTM to learn the overall relationship of feature sequences to predict DDIs [18]. Zheng et al. constructed a gene-drug pair sequence of length 2 and input it into the LSTM to predict drug-target interactions. Their results show that LSTM’s classification performance is better than other deep learning methods [19].

In Euclidean space, every pixel in an image can be regarded as a vertex in a graph, and each vertex is connected with a fixed number of adjacent pixel points. Convolutional neural network (CNN) can greatly speed up the training tasks related to images. Dhami et al. used CNN to predict DDIs directly from images of drug structures [20]. However, due to the inconsistency of the number of adjacent points of each vertex in the graph data structure, the image convolution operation is not applicable in non-Euclidean space. Kipf and Welling proposed a graph convolutional neural network (GCN), which extended convolution to the non-Euclidean space [21]. Feng et al. proposed a DDIs predictor combining GCN and DNN, in which each drug was modeled as a node in the graph, and the interaction between drugs was modeled as an edge. Features were extracted from the graph by GCN and input into DNN for prediction [22]. Zitnik et al. proposed Decagon, a DDIs prediction model based on GCN and multimodal graph, which embedded the relationship between drugs, proteins, and side effects to provide more information [23]. In general, similar structures and properties of drugs are associated with similar drug side effects [24, 25]. Ma et al. encoded each drug into a node in the graph and the similarity between drugs was coded into an edge. A multi-view graph autoencoder (GAE) based on drug characteristics was used to predict DDIs [26].

Due to a large amount of diverse drug information data, DDI prediction in silico remains a challenge and there is still room for improvement in prediction performance. In 2010, the National Institute of Health (NIH) funded the Library of Integrated Network-based Cellular Signatures (LINCS) project. This project aims to draw a comprehensive picture of multilevel cellular responses by exposing cells to various perturbing agents [27]. The L1000 database of the LINCS project has collected millions of genome-wide expressions induced by 20,000 small molecular compounds on 72 cell lines [28]. Applying deep learning, the L1000 database has previously been used to predict adverse drug reactions [29], drug pharmacological properties [30, 31], and drug-protein interaction [32]. However, whether this unified and comprehensive transcriptome data resource can be used to build a better DDI prediction model is still unclear. In this study, based on drug-induced transcriptome data in the L1000 database, we aim to explore DDI prediction by developing a new deep learning model with GCAN and LSTM.

## Results

### GCAN embedding of drug-induced transcriptome data

**Fig. 1:**
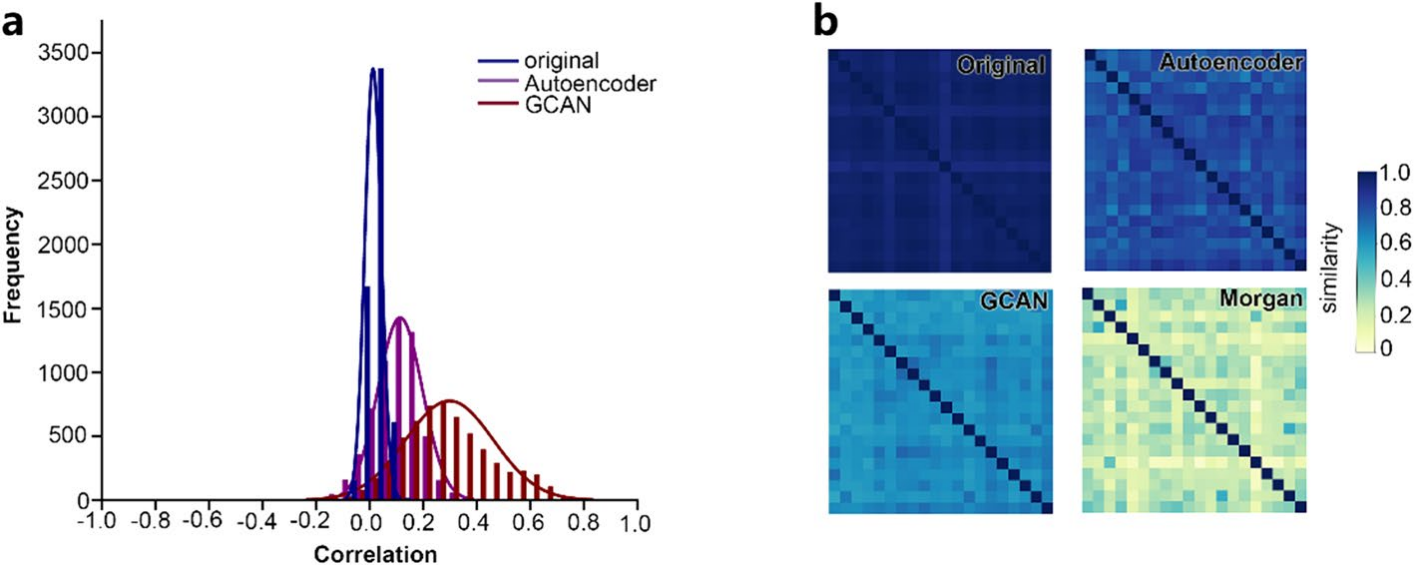
The Embedding of Drug-Induced Transcriptome Data by GCAN. **a** The correlation analysis between drug-induced transcriptome data, embedded features (autoencoder and GCAN) and drug structure. **b** The heat map of drug similarity

Since the original drug-induced transcriptome data contains technical noise, the correlation observed between drug-induced transcriptome data and drug structure is very low. In order to reduce the impact of noise, the drug-induced transcriptome data was embedded before building a DDI prediction model. To establish a stronger relationship between the drug structure and drug-induced transcriptome data, we used both the structure information of drugs and the similarity information between drugs in the process of embedding with GCAN. As shown in Fig. 1a, without embedding, the Pearson correlation coefficients between drug-induced transcriptome data and drug structure are 0. After the GCAN embedding, the majority of Pearson correlation coefficients between GCAN embedded features and drug structures increased to 0.25. In addition, 20 drug molecules were randomly selected to calculate their similarity based on different features. The heat maps of similarity between those drugs in Fig. 1b show that overall relationships between GCAN embedded features and drug structures are improved. We also tried to only use the structure information of drugs to embed drug-induced transcriptome data through an autoencoder network. Compared with GCAN embedded features, we observed less improvement in the correlation between the autoencoder embedded drug features and the drug structure (Fig. 1a, b).

### DDI prediction with GCAN embedded features

To explore whether GCAN embedded features can improve DDI prediction, we compared different drug features as input in various machine learning methods [15–17], and the prediction performance was evaluated through fivefold cross-validation. Results are summarized in Table 1. In contrast to the original drug-induced transcriptome data, GCAN embedded features significantly improved DDI performance in all models. In the traditional multi-label classification models such as MLKNN and Random forest, GCAN embedded feature led to larger improvement than autoencoder embedded features. The macro-F1 and macro-precision between GCAN embedded features and autoencoder embedded features for DDI prediction are not significantly different in the DNN model, but GCAN embedded features have a better DDI prediction macro-recall.

**Table 1:** DDI prediction performance of various machine learning models with different drug features as input. The p value compared with using GCAN features is added in brackets

Method	Feature	Macro-F1	Macro-recall	Macro-precision
DNN	Original	90.1% ± 1.9% (0.001)	90.7% ± 1.8% (0.0051)	91.3% ± 2.3% (0.009)
	Autoencoder	91.3% ± 0.7% (0.0655)	90.8% ± 0.9% (0.0223)	93.2% ± 1.1% (0.6219)
	GCAN	**93.3% ± 1.4% (–)**	**93.9% ± 1.7% (–)**	**93.7% ± 1.4% (–)**
Random forest	Original	40.7% ± 1.8% (4E − 05)	35.7% ± 1.5% (4.3E − 05)	58.6% ± 1.4% (0.0008)
	Autoencoder	45.2% ± 2% (0.0004)	39.9% ± 1.9% (0.0004)	62.9% ± 2.3% (0.001)
	GCAN	**57.6% ± 3% (–)**	**51.6.9% ± 2.9% (–)**	**75.7% ± 4.2% (–)**
MLKNN	Original	40.5% ± 1.2% (1.2E − 05)	34.7% ± 1.1% (1E − 05)	54.9% ± 2.4% (2.9E − 05)
	Autoencoder	51.5% ± 1.5% (5.5E − 05)	46.5% ± 1.9% (0.0001)	63.5% ± 2% (6.6E − 06)
	GCAN	**74.3% ± 2.1% (–)**	**70.3% ± 1.9% (–)**	**83.4% ± 2.2% (–)**
BRkNNaClassifier	Original	29.9% ± 1.7% (1E − 05)	23.4% ± 1.5% (9E − 06)	52.2% ± 2.8% (4.2E − 05)
	Autoencoder	39.1% ± 1.3% (4.4E − 05)	32.3% ± 1.3% (2.7E − 05)	59.2% ± 2.1% (0.0003)
	GCAN	**67.5% ± 2.4% (–)**	**61.1% ± 2.4% (–)**	**83.4% ± 3.3% (–)**

Bold indicates the best prediction performance

**Fig. 2:**
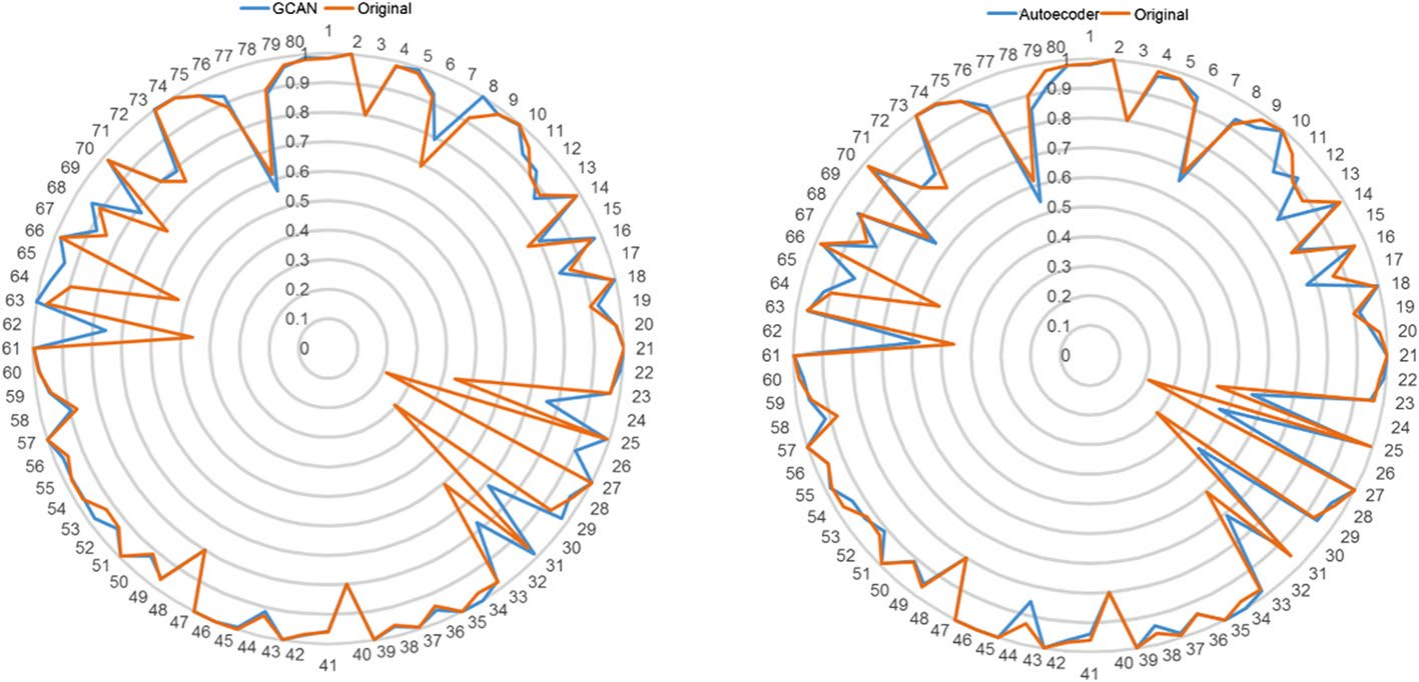
DDI prediction F1-score for each DDI type with DNN

To further evaluate the performance of GCAN embedded features, we examined the results of the DNN model under each DDI type. Compared with the original drug-induced transcriptome data, comparable or better classification F1-score is observed for 52 out of 80 DDI types when using GCAN embedded features, and for 41 out of 80 DDI types when using autoencoder embedded features (Fig. 2).

### Further improve DDI prediction with LSTM

DDIs often involve one drug changing the pharmacological effect of another [33], so it may be better to predict DDIs by treating the two drugs as a sequence. However, the DNN-based methods reported above simply combined the two drugs after feature extraction, without considering the sequence relationship between the drugs [15–17]. For this reason, we used LSTM to model this sequence relationship (For more details, see Additional file 1: Fig. S3 and Table S5). Experimental results in Table 2 show that LSTM is superior to DNN in macro-F1 or macro-recall for both the original drug-induced transcriptome data and embedded drug features. GCAN embedded drug features plus LSTM model has better prediction performance with a macro-F1 of 95.3% ± 1.5%, macro-precision of 94.6% ± 1.9%, and macro-recall of 96.6% ± 1.3% (Table 2).

**Table 2:** Comparison of DDIs prediction performance on LSTM and DNN model. The p value compared with LSTM is added in brackets

Feature	Method	Macro-F1	Macro-recall	Macro-precision
Original	DNN	90% ± 1.9% (0.0008)	90.7% ± 1.8% (0.0007)	91.3% ± 2.3% (0.0056)
	LSTM	**94.2% ± 1.9% (–)**	**95.5% ± 1.9% (–)**	**93.5% ± 1.9% (–)**
Autoencoder	DNN	91.2% ± 0.7% (0.086)	90.8% ± 0.9% (0.0013)	**93.2% ± 1.1% (0.0445)**
	LSTM	**92.5% ± 1.5% (–)**	**95.2% ± 1.6% (–)**	90.8% ± 1.6% (–)
GCAN	DNN	93.3% ± 1.4% (0.004)	93.9% ± 1.7% (0.008)	93.7% ± 1.4% (0.12)
	LSTM	**95.3% ± 1.5% (–)**	**96.6% ± 1.3% (–)**	**94.6% ± 1.9% (–)**

Bold indicates the best prediction performance

### DDI prediction performance in other cell lines and on other DDI databases

The above analysis demonstrates that the GCAN embedded features plus LSTM model is the best strategy for DDI prediction. In order to further validate its performance for DDIs across different cell lines, we processed the drug-induced transcriptome data of A357, A549, HALE, and MCF7 cells by GCAN, and compared the DDI prediction performance of these GCAN embedded features and original drug-induced transcriptome data within DNN vs LSTM based models. Table 3 shows the macro-F1, macro-recall and macro-precision indicators of GCAN embedded features for all four cell lines outperform the original drug-induced transcriptome data in both deep learning models, proving that GCAN embedded features are more suitable for DDI prediction. Additionally, when the LSTM model surpasses the DNN in terms of DDI prediction performance, it means that the LSTM model is better at learning potential drug relationships than the traditional DNN. Furthermore, GCAN embedded features plus LSTM achieve the best DDI prediction performance for all four cell lines. The findings also support that the proposed GCAN embedded features plus LSTM model can improve large-scale drug-induced transcriptome data analysis for DDI prediction.

**Table 3:** Comparison of model performance in other cell lines. The p value compared with GCAN + LSTM is added in brackets

Cell	Method	Macro-F1	Macro-recall	Macro-precision
A357	Original + DNN	85.3% ± 3% (0.001)	86.9% ± 3.5% (0.0003)	86.4% ± 2.8% (0.005)
	GCAN + DNN	88.8% ± 2% (0.03)	89.9% ± 2% (0.035)	89.8% ± 2.1% (0.029)
	Original + LSTM	89.2% ± 2.7% (0.005)	90.5% ± 3.6% (0.012)	89.5% ± (0.004)
	GCAN + LSTM	**92.8% ± 2.5% (–)**	**94.4% ± 2.7% (–)**	**92.4% ± 2.4% (–)**
A549	Original + DNN	87.4% ± 1.2% (0.001)	88.2% ± 1.4% (0.001)	89% ± 1.3% (0.01)
	GCAN + DNN	89.8% ± 1.6% (3.7E − 05)	90% ± 2.1% (0.0002)	91.5% ± 1.9% (0.112)
	Original + LSTM	90.4% ± 1.1% (0.003)	91.9% ± 1.5% (0.011)	90.2% ± 0.8% (0.63)
	GCAN + LSTM	**92.7% ± 1.6% (–)**	**94.1% ± 2.4% (–)**	**92.3% ± 1.6% (–)**
HA1E	Original + DNN	86.4% ± 2% (0.0007)	87.9% ± 1.9% (0.0004)	87.4% ± 2.2% (0.003)
	GCAN + DNN	90.8% ± 1.4% (0.001)	91.3% ± 1.7% (0.002)	91.8% ± 1.4% (0.002)
	Original + LSTM	91.6% ± 1.3% (0.012)	92.3% ± 1.7% (0.01)	91.9% ± 1.1% (0.021)
	GCAN + LSTM	**94.5% ± 0.8% (–)**	**95.9% ± 0.7% (–)**	**94.1% ± 0.9% (–)**
MCF7	Original + DNN	88.9% ± 1.3% (0.001)	89.3% ± 1.4% (0.001)	90.5% ± 2.2% (0.021)
	GCAN + DNN	92.9% ± 1.2% (0.005)	93.5% ± 1.4% (0.0004)	93.5% ± 1.2% (0.741)
	Original + LSTM	93% ± 1.1% (0.01)	94.9% ± 1.8% (0.011)	92.3% ± 1.1% (0.104)
	GCAN + LSTM	**94.8% ± 1.6% (–)**	**96.7% ± 1.9% (–)**	**93.6% ± 1.6% (–)**

Bold indicates the best prediction performance

In addition, we have replicated our model on two datasets (DS1 and DS2) from Rohani and Eslahchi’s report [17]. As compared with the prediction method used in their report, our model achieves better prediction performance (Additional file 1: Table S3 and Table S4).

### Advantage of GCAN embedded features for DDI prediction

**Fig. 3:**
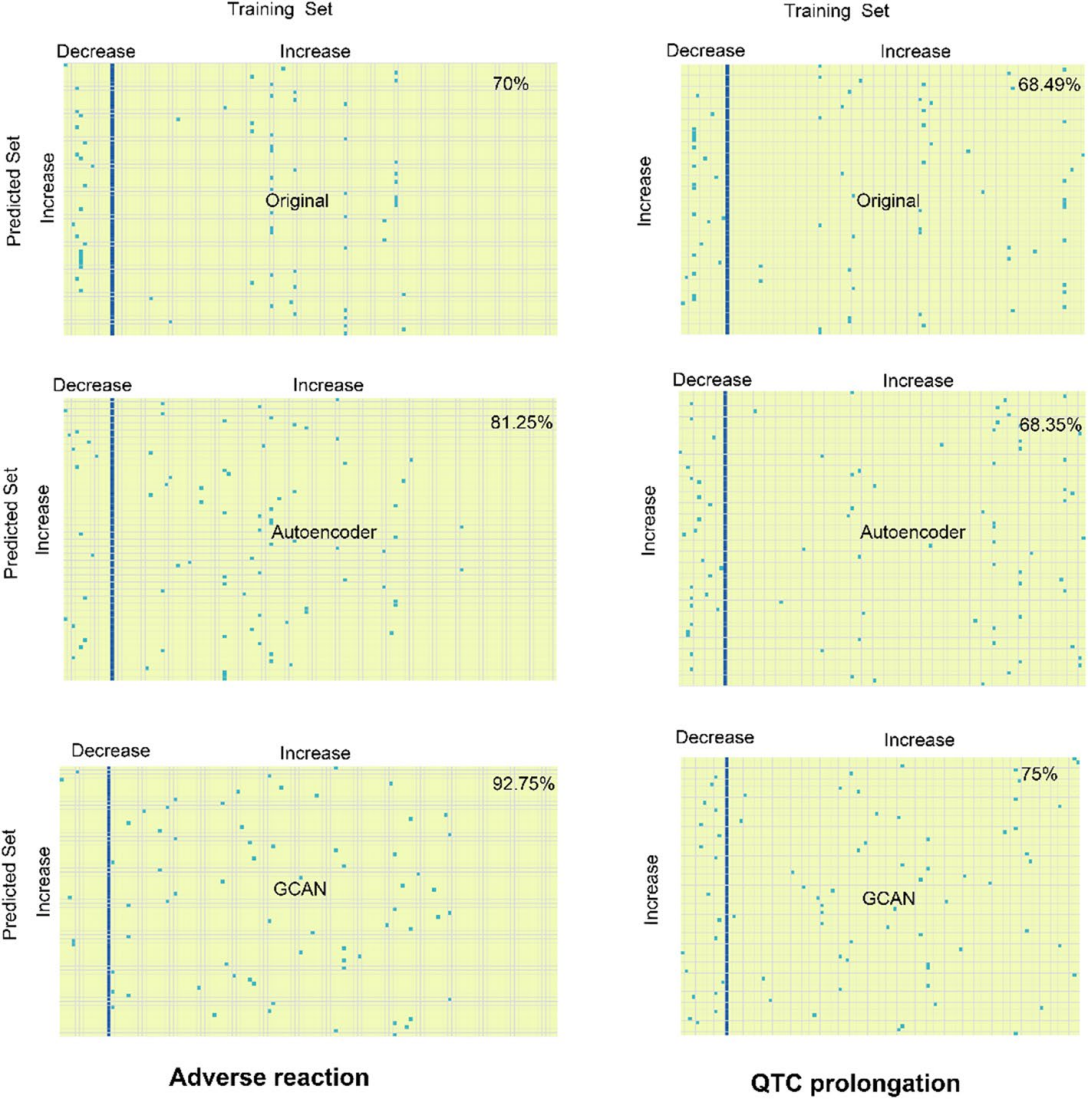
Similarity between drugs predicted to increase adverse reaction (with *cabergoline*) or QTC prolongation (with *amodiaquine*) and drugs (increased or decreased adverse reaction with *cabergoline*) or drugs (increased or decreased QTC prolongation with *amodiaquine*) of training set. The most similar drugs in each row are represented by blue-green dots

One of the fundamental assumptions in building a computational model for drug research is that the more similar the drugs are, the more similar their effects are [23], which is also the basic assumption in many DDI prediction studies. As a neural network is a black box, it is difficult to determine whether the knowledge learned by a neural network also aligns with this assumption. Under this consideration, we explored the ability of our model to identify the most similar samples in the training set when predicting DDIs. In our training set, there are 12 drugs that interact with the drug *cabergoline* and can lead to decreased adverse reaction; and there are 114 drugs that can cause an increased adverse reaction. The number of drugs that interact with the drug *amodiaquine* and can lead to decreased QTC prolongation or increased QTC prolongation is 12 and 96, respectively. Using GCAN embedded features plus LSTM for DDI prediction, 92.75% of the drugs that are predicted to interact with *cabergoline* and increase adverse reaction are most similar to one of the drugs that are known to interact with *cabergoline* and increase adverse reaction in the training set (Fig. 3). However, using autoencoder embedded features or original drug-induced transcriptome data, this discrimination is much lower, 81.25% and 70% respectively. In addition, we found that the highest proportion of drugs that are predicted to interact with *amodiaquine* and increase QTC prolongation are most similar to one of the drugs that are known to interact with *amodiaquine* and increase QTC prolongation in the training set by using GCAN embedded features. Therefore, it indicates that GCAN embedded features may improve DDI prediction by increasing the differentiation between drugs and is more consistent with the basic assumptions used in the drug-related computational model.

### Validation of new DDI predictions with an application in diabetes

**Fig. 4:**
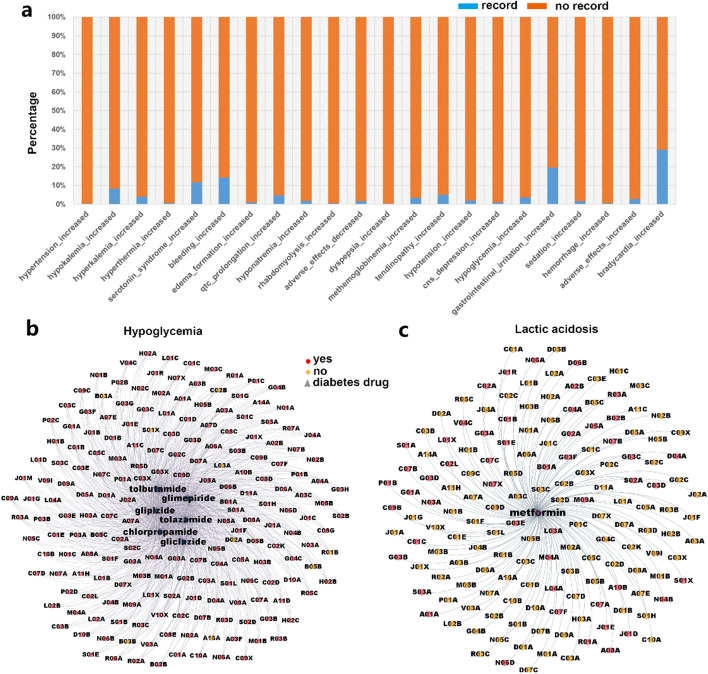
The new prediction DDIs. **a** New predicted DDIs are validated with latest DrugBank database. **b** Sulfonylurea hypoglycemic drugs interact with other disease drugs and cause hypoglycemia. **c** The interaction between metformin and other drugs of diseases leads to lactic acidosis. In the network diagram, the red circle indicates that DDIs can be explained through molecular mechanism, and the yellow circle indicates that DDIs cannot be explained, triangle for diabetes drugs

The next question that we aimed to answer is whether our proposed DDI prediction model can be used for the discovery of new DDIs. To find new DDIs, the entire DDI dataset (total 89,970 drug pairs) was input into the trained model to predict DDIs. After excluding the existing DDIs, a total of 21,670 new DDIs were predicted. Then we used the latest version of the DrugBank database (version 5.1.7) data released in April 2020 to verify the prediction results and found that 975 new DDIs were included in the latest DrugBank database (version 5.1.7) (Fig. 4a).

Cytochrome P450 enzyme (CYP450) is a key enzyme for drug metabolism in vivo. The inhibition of this enzyme’s activity will lead to drug accumulation and cause potential drug side effects [34, 35]. *Sulfonylurea* hypoglycemic drugs are mainly metabolized by CYP2C9 of CYP450 enzyme in the human liver. It has been reported that drugs with inhibition on CYP450, such as antibacterial drugs, antidepressants, and cardiovascular drugs, can interact with *sulfonylurea* hypoglycemic drugs, affecting the metabolism of *sulfonylurea* hypoglycemic drugs, and increase the risk of hypoglycemia [36]. Through our prediction model, we also identified new DDIs between *sulfonylurea* hypoglycemic drugs and antibacterial drugs (ATC code beginning with J01), antidepressants (ATC code beginning with N06), and cardiovascular drugs (ATC code beginning with C) can cause hypoglycemia. In addition, we also found that drugs indicated for many other diseases can interact with sulfonylureas hypoglycemic drugs to cause hypoglycemia. Online target prediction analysis [37] shows that almost all of these drugs may bind to the CYP450 enzyme (Fig. 4b).

*Metformin* has been used in the treatment of type 2 diabetes for more than 60 years and is still a first-line hypoglycemic drug widely used in the clinic. *Metformin* is not easily metabolized after entering the human body, 80–100% of *metformin* will be discharged from the renal tubules in the form of the prototype [38], thus the drugs that affect the renal function will affect the metabolism of *metformin*. Studies have shown that the elimination of *metformin* is mediated by the transporters MATE1, MATE2K, and OCT2 in the kidney [39]. *Cimetidine*, a drug for the treatment of peptic ulcers, can lead to *metformin* accumulation by competing with *metformin* for OCT2 or MATE1, which can result in a significant increase in the concentration of lactate in blood [40]. Our model also finds that many drugs that inhibit OCT2, MATE1, or MATE2K can interact with *metformin* and lead to lactic acidosis (Fig. 4c).

## Discussion

With the rapid development of high-throughput sequencing technology in recent years, multiple drug-induced transcriptome datasets have been accumulated in the LINCS L1000 database, which provides new mediums for characterizing drugs and new approaches for building predictive models for DDIs. The main contribution of this study is the development of a better deep-learning-based DDI prediction model using large-scale drug-induced transcriptome data. We utilized the information on chemical structures of drugs and the similarity between drug structures to embed the original drug-induced transcriptome data through GCAN. Our results show that GCAN embedded features is more effective for the prediction of DDIs, and the performance of DDI prediction is significantly improved in contrast to using original drug-induced transcriptome data in multiple machine learning methods. Several studies have reported that the DNN model based on drug structure data can significantly improve DDI prediction [15–17], but the prediction performances of other deep learning methods are still unclear. By comparing DNN and LSTM, we found that the macro-F1, macro-precision, and macro-recall predicted by LSTM is significantly higher than that of DNN. Finally, our proposed GCAN embedded features plus LSTM model significantly improves the prediction of DDIs based on drug-induced transcriptome data.

In addition, we verified some of the newly predicted DDIs by our model from two aspects. On the one hand, we searched the latest DrugBank database (version 5.1.7) and found that the number of newly recorded DDIs is predicted by our model. On the other hand, we analyzed the potential molecular mechanisms of newly predicted DDIs of antidiabetic agents through online drug-target interaction prediction [38]. We found that the predicted interacting drugs of *sulfonylureas* can cause hypoglycemia and interacting drugs of *metformin* can cause lactic acidosis, both of which have effects on the proteins involved in the metabolism of *sulfonylureas* and *metformin* in vivo. These results demonstrate that our model is superior in the prediction of DDIs.

With the development of drug delivery technology, more attention has been focused on macromolecule drug [41, 42]. One of the obvious characteristics of macromolecular drugs is the larger molecular structure. Therefore, the current approach in characterizing structures of small molecules is not suitable to accurately describe the structure of large molecules, and the existing DDI prediction model based on small molecular structures cannot predict DDIs of large molecular drugs. In contrast, drug-induced transcriptome data is the response of cells to drug-related properties, it can well characterize the macromolecular drugs. Thus, using drug-induced transcriptome data is a promising approach toward building an accurate macromolecular drug-related DDIs prediction model. However, since the small molecular structure information is used to embed drug-induced transcriptome data, the model proposed here cannot be directly used to predict DDIs related to macromolecular drugs. In future work, one potential solution is to use the target gene [43, 44], side effects [45], and Gene Ontology information [46] of drugs to embed the drug-induced transcriptome data with GCAN.

## Conclusions

In this paper, we propose GCAN embedded features plus LSTM model for the prediction of DDIs on drug-induced transcriptome data. Through evaluation of different models, the proposed model is demonstrated to significantly improve the prediction performance of DDIs. With a deep analysis of drugs interacting with *sulfonylureas* and *metformin*, we show that the new DDIs predicted by our model have good molecular mechanism support and many of the predicted DDIs are listed in the latest DrugBank library (version 5.1.7). These results indicate that our model has the potential to provide accurate guidance for drug usage.

## Methods

### Extraction of drug features

We used the LINCS L1000 dataset that includes ~ 205,034 gene expression profiles perturbed by more than 20,000 compounds in 71 human cell lines. LINCS L1000 is generated using Luminex L1000 technology where the expression levels of 978 landmark genes are measured by fluorescence intensity. The LINCS L1000 dataset provides five different levels of data depending on the stage of the data processing pipeline. Level 1 dataset contains raw expression values from the Luminex 1000 platform; Level 2 contains the gene expression values of 978 landmark genes after deconvolution; Level 3 provides normalized gene expression values for the landmark genes as well as imputed values for an additional ~ 12,000 genes; Level 4 contains z-scores relative to all samples or vehicle controls in the plate; Level 5 is the expression signature genes extracted by merging the z-scores of replicates. We utilized the Level 5 dataset marked as exemplar signature, which is relatively more robust, thus a reliable set of differentially expressed genes (DEGs). We took the subtraction expression values of 977 landmark genes between drug-induced transcriptome data and their untreated controls, resulting in a vector of 977 in length to represent each drug. The drug-induced transcriptome data in the PC3 cell line was used to build and evaluate the model. Data from the A375, A549, HA1E, or MCF7 cell lines were used to further validate the model. The reason we picked up data on these cells is that there are enough drug-induced transcriptome data on these cells.

### Preparation of the gold standard DDI dataset

The reported total of 2,723,944 DDIs described in the form of sentences were downloaded from DrugBank (version 5.1.4). Drugs with more than one active ingredient, proteins, and peptidic drugs were not considered in this study, and drugs with no transcriptome data in the PC3 cell line from the L1000 dataset were also excluded. Since our model was trained and evaluated with fivefold cross-validation, adverse DDI types with less than 5 drug pairs in them were excluded. Finally, a total of 89,970 DDIs were classified into 80 DDI types and used to construct the DDI prediction model (For more information, see Additional file 1: Table S1).

### Proposed deep learning model for DDI prediction

**Fig. 5:**
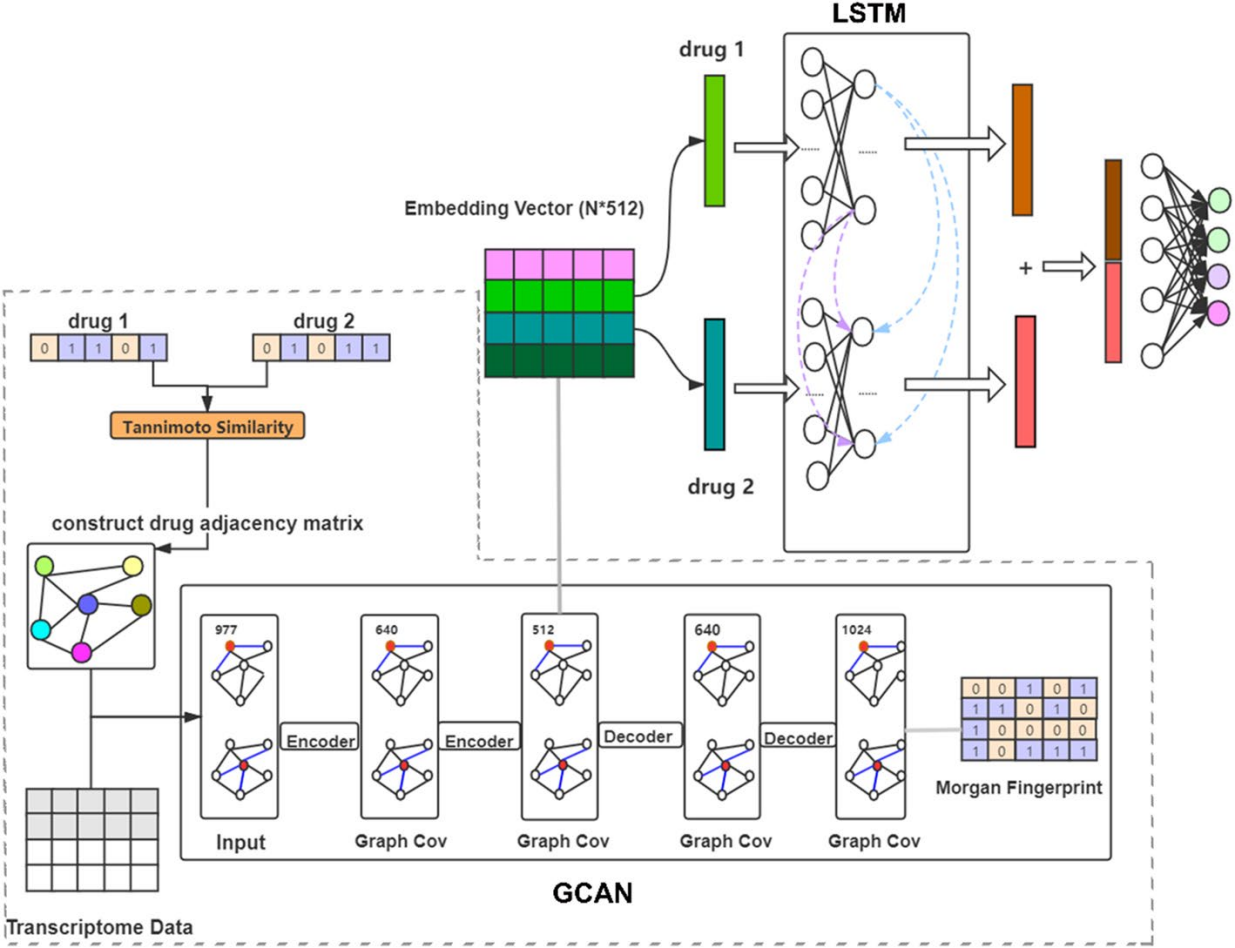
GCAN plus LSTM model for DDI prediction

The DDI prediction model proposed in this study consists of two parts (Fig. 5). First, a GCAN is used to embed the drug-induced transcriptome data. Then the embedded drug features are input into LSTM networks for DDIs prediction.

In the GCAN graph [47], each node represents a single drug which connected to other 40 drugs with the most similar chemical structure described by the Morgan fingerprint. The Tanimoto coefficient [48] is calculated to measure the similarity between drug structures. After the similarity matrix between drug structures is built, a maximum of 40 values are retained in each row and the rest are replaced by 0. Then each row of this similarity matrix is normalized to represent the weight of connecting edges between drugs. The GCAN network combined features information of each node and its most similar nodes by multiplying the weights of the graph edges, and then we use sigmoid or tanh function to update the feature information of each node. The whole GCAN network is divided into two parts: encoder and decoder, summarized in Additional file 1: Table S2. The encoder has three layers with the first layer being the input of drug features, the second and third are the coding layers (dimensions of the three layers are 977, 640, 512 respectively). There are also 3 layers in the decoder where the first layer is the output of the encoder, the second layer is the decoding layer, and the last layer is the output of the Morgan fingerprint information (three layers of the drug features dimension are 512, 640, 1024 respectively). After obtaining the output of the decoder, we calculate the cross-entropy loss of the output and Morgan fingerprint information as the loss of the GCAN and then use backpropagation to update the network parameters (learning rate is 0.0001, L2 regular rate is 0.00001). Every layer except the last layer uses the tanh activation function and the dropout value is set to 0.3. The GCAN output is the embedded data to be used in the prediction model.

Since DDI often involves one drug causing a change in the efficacy and/or toxicity of another drug, treating two interacting drugs as sequence data may improve DDI prediction. Thus, we choose to construct an LSTM model by stacking the embedded features vectors of two drugs into a sequence as the input of LSTM. Optimization of the LSTM model in terms of the number of layers and units in each layer by using grid search, and is shown in Additional file 1: Fig. S1. Finally, the LSTM model in this study has two layers, each layer has 400 nodes, and the forgetting threshold is set to 0.7. In the training process, the learning rate is 0.0001, the dropout value is 0.5, the batch value is 256, and the L2 regular value is 0.00001.

We also perform DDI prediction using other machine learning methods including DNN, Random Forest, MLKNN, and BRkNNaClassifier. By using grid search, the DNN model is optimized in terms of the number of layers and nodes in each layer. It is shown in Additional file 1: Fig. S2. The parameters of Random Forest, MLKNN, and BRkNNaClassifier models are the default values of Python package scikit-learn [49].

### Evaluation metrics

The model performance is evaluated by fivefold cross-validation using the following three performance metrics:

1 $$Marco - recall = \frac{{\mathop \sum \nolimits_{{i = 1}}^{n} \frac{{TP_{i} }}{{TP_{i} + FN_{i} }}}}{n}$$

2 $$Marco - precision = \frac{{\mathop \sum \nolimits_{{i = 1}}^{n} \frac{{TP_{i} }}{{TP_{i} + FP_{i} }}}}{n}$$

3 $$Marco - F1 = \frac{{2\left( {Marco - precision} \right)\left( {Marco - recall} \right)}}{{\left( {Marco - precision} \right) + \left( {Marco - recall} \right)}}$$

where TP, TN, FP, and FN indicate the true positive, true negative, false positive, and false negative, respectively, and *n* is the number of labels or DDI types. Python package scikit-learn [49] is used for the model evaluation.

### Correlation analysis

In this study, the drug structure is described with Morgan fingerprint. The Tanimoto coefficient is calculated to measure the similarity between drug structures. The transcriptome data or GCAN embedded data are all floating-points and the similarity can be calculated using the European distance as follow:

4 $${\text{drug}}\_{\text{similarity}}\left( {{\text{X}},{\text{Y}}} \right) = \frac{1}{{\mathop \sum \nolimits_{{{\text{i}} = 1}}^{{\text{d}}} \left( {{\text{X}}_{{\text{i}}} - {\text{Y}}_{{\text{i}}} } \right)^{2} + 1}}$$

where X and Y represent transcriptome data, *d* stands for characteristic dimension.

To measure the relationship between different characteristic vectors of drugs, the Pearson correlation coefficients are calculated. First, the similarity between drugs is calculated with different characteristics of drugs. Then the similarity between drugs is used to represent drugs, and the Pearson correlation coefficients is calculated with this new characteristic as follows:

5 $${\mathrm{\rho }}_{\mathrm{x},\mathrm{y}}=\frac{\mathrm{E}\left(\mathrm{x}\mathrm{y}\right)-\mathrm{E}(\mathrm{x})\mathrm{E}(\mathrm{y})}{{\mathrm{\sigma }}_{\mathrm{x}}{\mathrm{\sigma }}_{\mathrm{y}}}$$

where *E* is the mathematical expectation, *σ* is the variance, and *x* and *y* are two different drug vectors.

## Supplementary Information

**Additional file 1**: Fig. S1 Optimization of the LSTM model in terms of the number of layers and nodes in each layer. Fig. S2 Optimization of the DNN models with three different drug features in terms of the number of layers and nodes in each layer. Fig. S3 DDI prediction with LSTM. Table S1 Preparation of the Gold Standard DDI Dataset. Table S2 Optimal parameters of GCAN. Table S3 Performance comparison on DS1. Table S4 Performance comparison on DS2. Table S5 Performance on different orders of the drugs.

## Data Availability

The datasets generated and analyzed during the current study and the code are freely available at https://github.com/BDAII/DDI.

## Abbreviations

DDI: Drug-drug interaction
NIH: National Institute of Health
LINCS: Library of Integrated Network-based Cellular Signatures
GCAN: Graph convolutional autoencoder network
LSTM: Long short-term memory networks
CYP450: Cytochrome P450 enzymes
DEGs: Differentially expressed genes
DNN: Deep neural network
