# Supplementary material for: Novel deep learning-based transcriptome data analysis for drug-drug interaction prediction with an application in diabetes
Source: BMC Bioinformatics. 2021 Jun 11;22:318. doi: 10.1186/s12859-021-04241-1 (PMC8194123; doi:10.1186/s12859-021-04241-1)
Supplement: Supplementary file 1 — Additional file 1: Fig. S1 Optimization of the LSTM model in terms of the number of layers and nodes in each layer. Fig. S2 Optimization of the DNN models with three different drug features in terms of the number of layers and nodes in each layer. Fig. S3 DDI prediction with LSTM. Table S1 Preparation of the Gold Standard DDI Dataset. Table S2 Optimal parameters of GCAN. Table S3 Performance comparison on DS1. Table S4 Performance comparison on DS2. Table S5 Performance on different orders of the drugs. [file 12859_2021_4241_MOESM1_ESM.pdf]

Additional file for

Novel deep learning-based transcriptome data analysis for drug-drug interaction prediction with an application in diabetes

Authors:

Qichao Luo[1,2†], Shenglong Mo[1†], Yunfei Xue[1†], Xiangzhou Zhang[1†], Yuliang Gu[1†], Lijuan Wu[1], Jia Zhang[3], Linyan Sun[4], Mei Liu[5*], Yong Hu[1*]

Affiliation of the authors:

[1]Big Data Decision Institute, Jinan University, Guangzhou, 510632, China.

[2]School of Management, Jinan University, Guangzhou, 510632, China.

[3]Department of Geriatrics, The First Affiliated Hospital of Chongqing Medical University, Chongqing, 400016, China

[4]Xi'an Hospital of Traditional Chinese Medicine, Xi'an, 710021, China.

[5]Division of Medical Informatics, Department of Internal Medicine, University of Kansas Medical Center, Kansas City, KS, 66160, USA

†: **Equal contribution**

[*]**Corresponding author：Mei Liu, PhD**. E-mail: meiliu@kumc.edu; Yong Hu, PhD. E-mail: yonghu@jnu.edu.cn

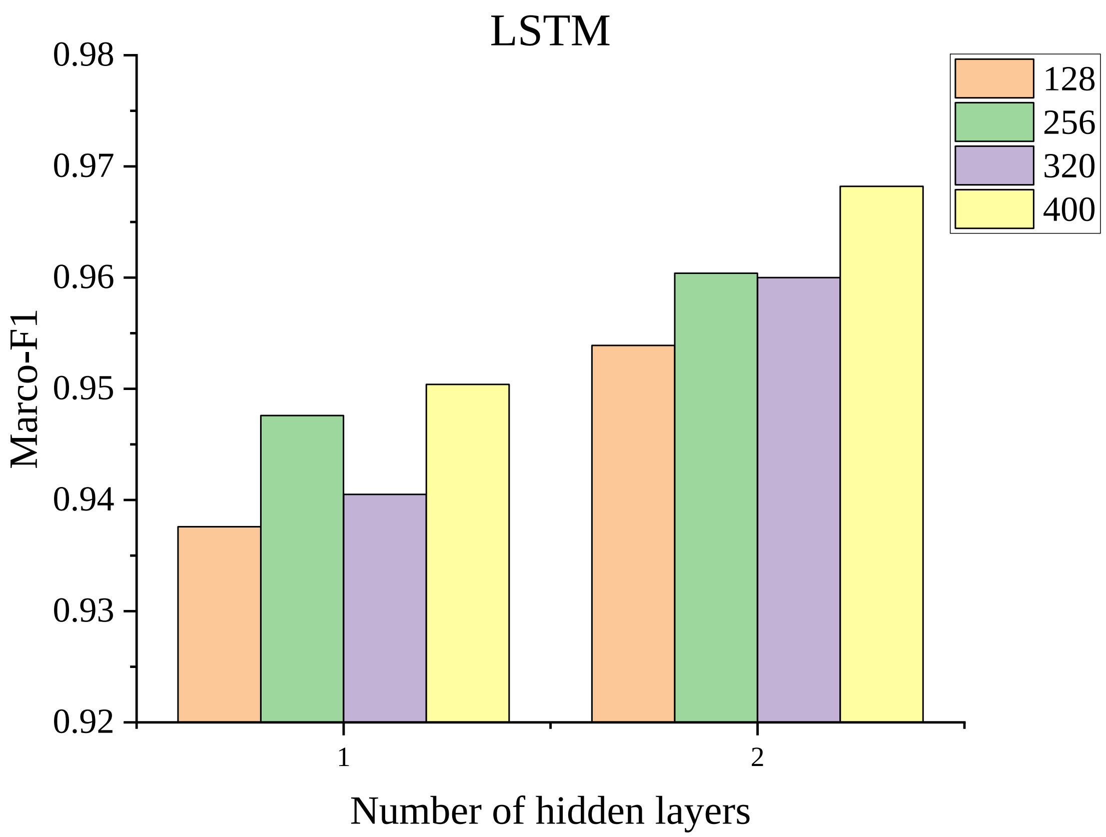

Fig. S1 Optimization of the LSTM model in terms of the number of layers and nodes in each layer. In order to optimize the architecture, we tested 128, 256, 320 and 400 nodes with 1 to 2 hidden layers.

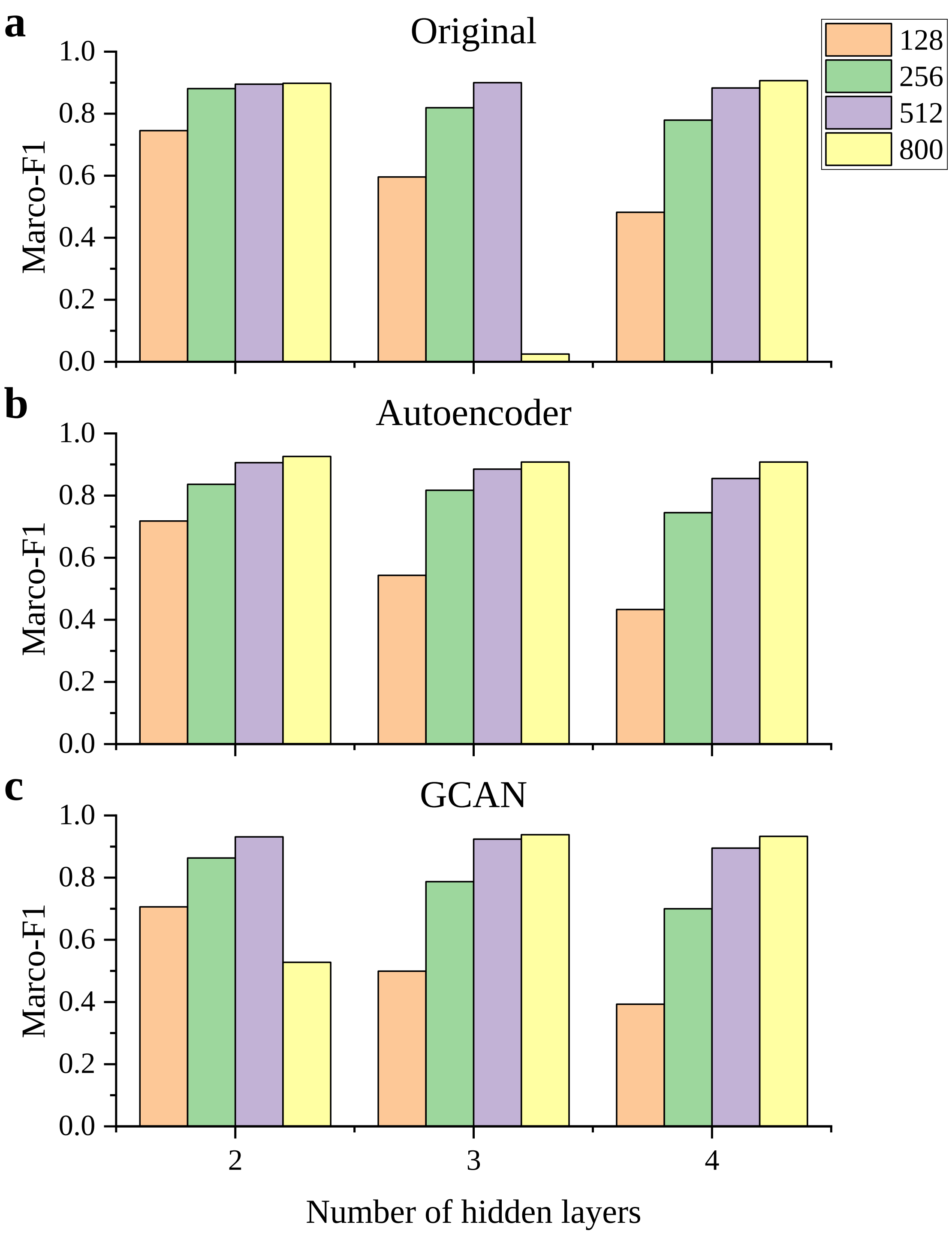

Fig. S2 Optimization of the DNN models with three different drug features in terms of the number of layers and nodes in each layer. a) Original drug-induced transcriptome data features. b) Autoencoder embedded features. c) GCAN embedded features. In order to optimize the architecture, we tested 128, 256, 512 and 800 nodes with 2 to 4 hidden layers.

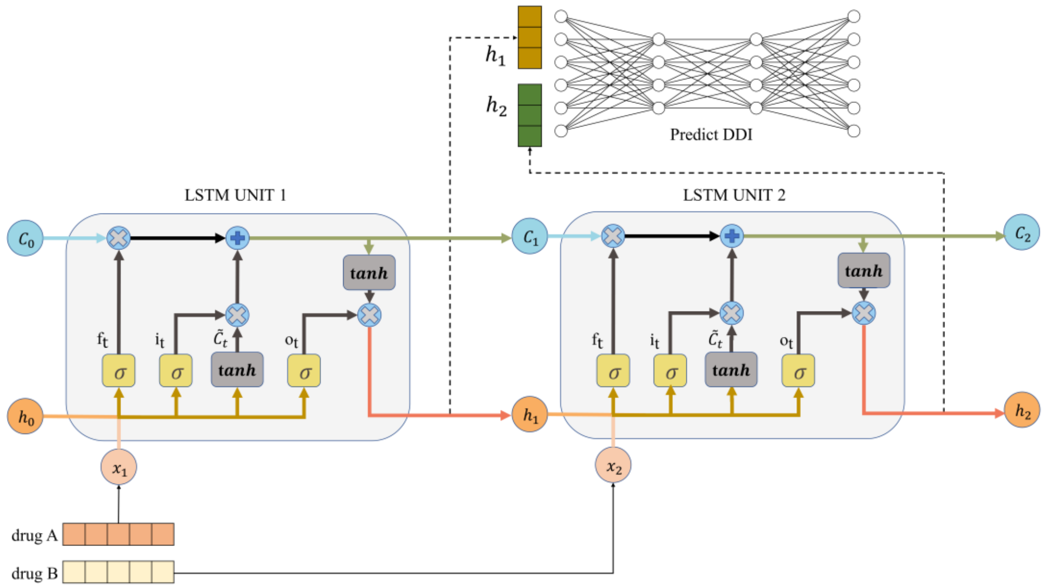

Fig. S3 DDI prediction with LSTM. In each LSTM unit, it contains $\text{forget}_{\text{gate}}$ (f_t), $\text{input}_{\text{gate}}$ (i_t), $\text{output}_{\text{gate}}$ (o_t). σ means sigmoid function.

DDIs are often caused by the correlation between the two drugs, such as antidepressant drugs combined with sulfonylurea hypoglycemic drugs can lead to hypoglycemia. Therefore, if we regard DDIs as semantics, then the semantics should be determined by the relevant features in the two drug eigenvectors.

In LSTM, the length of the sequence data is 2. Each element in the sequence data corresponds to the eigenvector of a drug. The order of the sequence data is determined by the order of the two drugs recorded in the DrugBank database. During training, drug A is input into UNIT 1 and transmits part of its characteristics to UNIT2 through mechanisms such as the $\text{forget}_{\text{gate}}$ (f_t) of LSTM. At this time, UNIT 2 contains part of the information of drug A and whole information of drug B, and finally obtains the final features through LSTM mechanisms like UNIT 1, so it can be seen that the final feature (h_2) includes the features of drug A and drug B. In the following prediction, we concatenated the final feature (h_2) and hidden state of UNIT 1 (h_1) to predict the DDI between drug A and drug B.

Table S1 Preparation of the Gold Standard DDI Dataset. For the labels of DDIs, we downloaded the descriptions of DDIs from the DrugBank database. The forms of descriptions, for an example, are like "The risk or severity of QTC prolongation can be increased When #drugA is combined with #drugB", we can extract the keywords of the description, such as "qtc prolongation", "increased", so the interaction between drug A and drug B is labeled "qtc_prolongation_increased". Each drug pair may have multiple types of interactions, causing it to belong to multiple labels.

	Number of remaining DDIs	Number of DDI types	Description of exclusion criteria
Initial DDIs	2,723,944	93	--
Exclude_1	90,661	93	Drugs with more than one active ingredient
Exclude_2	89,978	93	Proteins and peptidic drugs; Drugs with no transcriptome data in the PC3 cell line from the L1000 dataset
Exclude_3	89,970	80	Adverse DDI types with less than 5 drug pairs
Final DDIs	89,970	80	--

Table S2 Optimal parameters of GCAN

The parts of GCAN	Layer	Number of nodes
Encoder	1	977
	2	640
	3	512
Decoder	1	512
	2	640
	3	1024

Table S3 Performance comparison on DS1

Method	AUC	F-measure	Recall	Precision
RF	0.83	0.666	0.738	0.607
LR	0.941	0.812	0.81	0.818
Adaptive boosting	0.722	0.558	0.572	0.546
LDA	0.935	0.801	0.8	0.803
QDA	0.857	0.751	0.912	0.638
KNN	0.73	0.08	0.062	0.098
Substructure-based label propagation model	0.937	0.804	0.797	0.811
Side-effect-based label propagation model	0.936	0.806	0.793	0.82
Offside-effect-based label propagation model	0.937	0.809	0.795	0.823
Vilar's substructure-based model	0.936	0.804	0.797	0.812
Classifier ensemble method	0.956	0.836	0.827	0.843
Weighted average ensemble method	0.948	0.831	0.835	0.826
NDD	0.954	0.835	0.836	0.833
**Ours**	**0.9992**	**0.9993**	**0.9992**	**0.9994**

Table S4 Performance comparison on DS2

Method	AUC	F-measure	Recall	Precision
RF	0.982	0.747	0.713	0.785
LR	0.911	0.318	0.397	0.268
Adaptive boosting	0.904	0.266	0.359	0.211
LDA	0.894	0.295	0.407	0.231
QDA	0.926	0.174	0.875	0.096
KNN	0.927	0.602	0.445	0.932
Substructure-based label propagation model	0.788	0.294	0.537	0.197
Vilar's substructure-based model	0.81	0.312	0.479	0.232
Classifier ensemble method	0.936	0.553	0.689	0.462
Weighted average ensemble method	0.646	0.15	0.226	0.118
NDD	0.994	0.825	0.804	0.847
**Ours**	**0.9994**	**0.9993**	**0.9994**	**0.9993**

Table S5 Performance on different orders of the drugs. The p value compared with Reverse-LSTM is added in brackets.

Feature	Method	Macro-F1	Macro-recall	Macro-precision
GCAN	DNN	93.3% ± 1.4% (4.1E-5)	93.9% ± 1.7% (0.0088)	93.7% ± 1.4% (0.0148)
	LSTM	**95.3% ± 1.5% (0.4402)**	**96.6% ± 1.3% (0.851)**	**94.6% ± 1.9% (0.4551)**
	Reverse-LSTM	94.6% ± 1.6% (-)	95.6% ± 2.3% (-)	94.2% ± 1.2% (-)

In terms of whether the order of the features would affect the performance, we believe that the order of the drugs (features) in drug pair sequence does not affect the performance. To verify this assumption, we reverse the order of the drugs in drug pair sequence of the whole dataset and fix other settings to retrain the model (Reverse-LSTM). There is no significant difference between Reverse-LSTM and LSTM in all three metrics (Table S5), indicating that the order of drugs doesn't have a significant impact on the model performance. But both LSTM and Reverse-LSTM are better than DNN in all three metrics (see Table 2 and Table S5). The result indicates that LSTM module learned the association between the drugs in the sequence, that is the latent DDI semantic information, which can improve the performance of the model in predicting DDIs.
